# Synthesis and Biological Activity Evaluation of 2-Cyanopyrrole Derivatives as Potential Tyrosinase Inhibitors

**DOI:** 10.3389/fchem.2022.914944

**Published:** 2022-06-17

**Authors:** Ya-Guang Hu, Zhu-Peng Gao, Ying-Ying Zheng, Chun-Mei Hu, Jing Lin, Xiao-Zheng Wu, Xin Zhang, Yong-Sheng Zhou, Zhuang Xiong, Dao-Yong Zhu

**Affiliations:** ^1^ School of Pharmacy and State Key Laboratory of Applied Organic Chemistry, Lanzhou University, Lanzhou, China; ^2^ School of Biotechnology and Health Sciences, Wuyi University, Jiangmen, China; ^3^ Guangzhou Yuming Biologival Technology Co, LTD, Guangzhou, China

**Keywords:** tyrosinase, reversible inhibitor, mixed-type inhibitor, 2-cyanopyrrole, molecular docking

## Abstract

In order to find potential inhibitors of tyrosinase, two series of pyrrole derivatives A (1–17) and B (1–8) were synthesized and screened for their inhibitory activities on tyrosinase. Most of the 2-cyanopyrrole derivatives exhibited effective inhibitory activities. In particular, A12 exhibited the strongest inhibitory activities, with the IC_50_ values of 0.97 μM, which is ∼30 times stronger than the reference inhibitor kojic acid (IC_50_: 28.72 μM). The inhibitory mechanism analysis results revealed that A12 was a reversible and mixed-type inhibitor. Molecular docking experiments clarified the interaction between A12 with tyrosinase. Furthermore, A12 (100 μM) presented effective inhibitory effect on tyrosinase in B16 melanoma cells with inhibition of 33.48%, which was equivalent to that of Kojic acid (39.81%). Accordingly, compound A12 may serve as the lead structure for the further design of potent tyrosinase inhibitors. Molecular docking studies confirmed the interaction between the compound and tyrosinase.

## Introduction

The browning of food including fruits, vegetables, and beverages seriously threats the development of agriculture and the food industry (Chai et al., 2018). Recently, quality loss of food during postharvest handling and processing occur frequently in the food industry (Zhu et al., 2011). The recognized mechanism of the corresponding browning includes two types of procedures, namely, enzymatic and non-enzymatic browning (Todaro et al., 2011). Tyrosinase, a metalloenzyme containing dinuclear copper ions, is one of the key catalytic proteins in the process of enzymatic browning (Liu et al., 2012; Asthana et al., 2015; Chai et al., 2015; Xie et al., 2016). It is widely distributed in animals, insects, plants, and microorganisms and is a rate-limiting enzyme involved in two-step oxidation reactions including mono phenols into diphenols and o-diphenols into o-quinones, followed by further transformations into dark brown pigments (Ullah et al., 2016; Cai et al., 2019; Ielo et al., 2019; Zolghadri et al., 2019). For plant-derived foods, enzymatic browning would result in abnormal variations of color, flavor, and nutritional quality (Larik et al., 2017). In addition, it has been believed that excessively active tyrosinase leads to the accumulation of melanin in the human skin, which results in various common skin diseases, such as freckles, melasma, and melanosis (Ullah et al., 2019a; Ullah et al., 2019b; Butt et al., 2019; Haldys and Latajka, 2019; Huang et al., 2019; Lee et al., 2019). In the biosynthesis pathway of melanin, tyrosinase oxidizes l-tyrosine into L-3,4-dihydroxyphenylalanine (l-DOPA), and l-DOPA into o-quinones (Akın et al., 2019; Ashooriha et al., 2019; Santi et al., 2019). Tyrosinase is also proven to be related to Parkinson’s and other neurodegenerative diseases (Ali et al., 2019). Moreover, tyrosinase plays a key role in the processes of cuticle formation in insects (Yu, 2003). Hence, developing tyrosinase inhibitors is very important in the agriculture and food industry, medicine, and cosmetic products.

Although hundreds of tyrosinase inhibitors have been obtained in the laboratory, only a few are sufficiently potent for practical use, such as arbutin, kojic acid, and ascorbic acid (Demirkiran et al., 2013; Crespo et al., 2019; Rodriguez et al., 2020; Wang et al., 2020). However, they still exhibit the defect of undesirable side effects, including cytotoxicity, dermatitis, and neurodegenerative disorders. Therefore, it is extremely urgent to develop alternatively safe and effective tyrosinase inhibitors.

Pyrrole, aromatic five-membered heterocyclic compounds containing nitrogen, have gotten increased attention due to their extensive biological, agrochemical, and pharmaceutical activities, as well as reactivity and stability (Deibl et al., 2015; Liao et al., 2015; Qin et al., 2015). Moreover, heterocyclic compounds containing nitrogen exhibit good water solubility and salt formation ability. Until now, more and more biologically active molecules were designed based on the pyrrole skeleton (Insuasty et al., 2010; Jamwal et al., 2013; Kumar et al., 2013; Ansari et al., 2017). Also, for searching tyrosinase inhibitors, N-heterocycle derivatives have received much attention.

Recently, we have reported some efficient synthetic methodologies of N-heterocycles, which are the key skeleton of many bioactive molecules (Mou et al., 2016; Zhang et al., 2017). As the systematic continuation of aforementioned research results, we synthesized two series of pyrrole derivatives and screened their tyrosinase inhibitory activity.

## Materials and Methods

### Materials

Mushroom tyrosinase (EC 1.14.18.1), L-3,4-dihydroxy phenylalanine (l-DOPA), and kojic acid were obtained from Sigma (St. Louis, MO, United States). Dimethyl sulfoxide (DMSO) of molecular biological grade was purchased from J&K Chemical Co., Ltd. (Shanghai, China). Fetal bovine serum (FBS) and Dulbecco’s Modified Eagle’s Medium (DMEM) were obtained from Gibco (Grand Island, NY, United States). The 3-(4,5-Dimethythiazol-2-yl)-2,5-diphenyl-tetra-zoliumbromide (MTT) dye was supplied by Sigma-Aldrich (St. Louis, MO, United States). All other reagents were of analytical grade.

### Instruments

^1^H and ^13^C NMR spectra were recorded in CDCl_3_ on 400/300 MHz instruments, and spectral data were reported in ppm. High-resolution mass spectral analysis (HRMS) data were measured on Apex II by means of the ESI technique.

### Synthesis of 2-Cyanopyrrole Derivatives A (1–17)

2-Cyanopyrrole derivatives A (1–17) were synthesized according to our previous article (Mou et al., 2016).

The general synthetic procedure for derivative A (1–12) is as follows. To a stirred mixture of aromatic olefin or alkyne (0.5 mmol, 1 equiv), DMF (15 equiv), TMSCN (trimethylsilanecarbonitrile, 5 equiv), and Cu(OTf)_2_ (0.2 equiv), was added DDQ (2 equiv) through a batch-wise mode (the first equiv was added 0.2 equiv/2 h for 5 times, and the second equiv of DDQ was then introduced at once) at 80°C under an argon atmosphere. Then, the reaction mixture was stirred for an additional 14 h. Upon the completion of the reaction (monitored by TLC), the mixture was quenched by the addition of water. The aqueous layer was extracted three times with EtOAc, and the combined organic layers were washed with brine, dried over sodium sulfate, evaporated to dryness, and purified by column chromatography to afford desired products A (1–12).

The general synthetic procedure for derivative A (13–17) is as follows. To a mixed solvent of n-heptane and N,N-diethylacetamide (v/v = 6:1, 1 ml), aromatic olefin (0.5 mmol, 1 equiv), N,N-disubstituted formamide (3 equiv), TMSCN (6 equiv), and Cu(OTf)_2_ (0.2 equiv) were successively added at 80°C under an argon atmosphere. Next, DDQ was added through a batch-wise mode (the first equiv was added 0.2 equiv/2 h five times, and the second equiv of DDQ was then introduced at once) at the same temperature. The reaction mixture was refluxed for an additional 14 h. Upon completion of the reaction (monitored by TLC), the reaction mixture was quenched by the addition of water. The aqueous layer was extracted three times with EtOAc, and the combined organic layers were washed with brine, dried over sodium sulfate, evaporated to dryness, and purified by column chromatography to afford desired products A (13–17).

### Synthesis of N-Heterocycle Derivatives B (1–8)

The general synthetic procedure for derivative B (1–2) is as follows. To a stirred mixture of A5 or A6 (1 mmol) in DMSO (10 ml), K_2_CO_3_ (2 mmol) and hydrogen peroxide (30% aqueous, 30 ml) were added. The mixture was stirred at room temperature for 8 h. When the substrate was completely disappeared (monitored by TLC), the reaction mixture was extracted with ether and dichloromethane (2: 1) three times. The combined organic layers were washed with saturated brine (50 ml), dried over sodium sulfate, filtered, and concentrated in vacuo. The residue was purified by column chromatography to give B (1–2).

The general synthetic procedure for derivative B (3–8) is as follows. To a stirred mixture of A6, A3, A5, A1, A9, or A2 (1 mmol) in ether (10 ml), lithium aluminum tetrahydride (1.2 mmol) was added slowly at 0°C. Then, the mixture was stirred at reflux for 3 h and quenched with water (10 ml). The mixture was extracted with ether three times. The combined organic layers were washed with brine (50 ml), dried over magnesium sulfate, and concentrated in vacuo to give crude primary amine 2. Then, the crude primary amine 2 (1 mmol), (Boc)_2_O (1.3 mmol), and Et_3_N (5 mmol) were added into methanol (10 ml) and refluxed for 20 min. The mixture was quenched with saturated aqueous ammonium chloride solution and extracted with diethyl ether three times. The combined organic layers were washed with brine (50 ml), dried over magnesium sulfate, and concentrated in vacuo. The crude residue was purified by column chromatography to afford B (3–8).

3-(3-Bromophenyl)-1-methyl-1H-pyrrole-2-carboxamide (B1). Amorphous solid; 39% yield; ^1^H NMR (400 MHz, CDCl_3_) δ 7.53–7.52 (m, 1H), 7.52–7.49 (m, 1H), 7.31–7.30 (m, 1H), 7.29–7.28 (m, 1H), 6.74 (d, J = 2.4 Hz, 1H), 6.09 (d, J = 2.4 Hz, 1H), 5.43–5.29 (m, 2H), 3.93 (s, 3H); ^13^C NMR (100 MHz, CDCl_3_) δ 163.45, 134.99, 131.81, 131.07, 127.98, 127.14, 121.46, 109.32, 109.28, 98.48, 37.31, and 37.27; HRMS (ESI) calcd for C_12_H_11_BrN_2_O [M + H]^+^: 279.0128, found 279.0124.

3-(4-Bromophenyl)-1-methyl-1H-pyrrole-2-carboxamide (B2). White solid; mp 172–174°C; 33% yield; ^1^H NMR (400 MHz, CDCl_3_) δ 7.52 (d, J = 8.4 Hz, 2H), 7.30 (d, J = 8.4 Hz, 2H), 6.73 (d, J = 2.4 Hz, 1H), 6.09 (d, J = 2.4 Hz, 2H), 5.48 (d, J = 9.6 Hz, 2H), 3.92 (s, 3H); ^13^C NMR (100 MHz, CDCl_3_) δ 163.52, 134.94, 131.79, 131.06, 127.95, 127.13, 121.44, 109.28, and 37.31; HRMS (ESI) calcd for C_12_H_11_BrN_2_O [M + H]^+^:279.0128, found 279.0123.

tert-Butyl ((3-(4-bromophenyl)-1-methyl-1H-pyrrol-2-yl)methyl)carbamate (B3). White solid; mp 109–113°C; 29% yield; ^1^H NMR (400 MHz, CDCl_3_) δ 7.48 (d, J = 8.4 Hz, 2H), 7.19 (d, J = 8.4 Hz, 2H), 6.64 (d, J = 2.4 Hz, 1H), 6.19 (d, J = 2.8 Hz, 1H), 4.59 (s, 1H), 4.39 (d, J = 5.2 Hz, 2H), 3.65 (s, 3H), and 1.46 (s, 9H); ^13^C NMR (100 MHz, CDCl_3_) δ 155.25, 135.28, 131.54, 129.55, 125.03, 123.82, 122.73, 119.67, 107.40, 79.65, 34.57, 34.10, and 28.33; HRMS (ESI) calcd for C_17_H_21_BrN_2_O_2_ [M + H]^+^: 365.0859, found 365.0853.

tert-Butyl ((3-(3-fluorophenyl)-1-methyl-1H-pyrrol-2-yl)methyl)carbamate (B4). White solid; mp 103–104°C; 44% yield; ^1^H NMR (400 MHz, CDCl_3_) δ 7.35–7.29 (m, 1H), 7.10–7.08 (m, 1H), 6.95–6.91 (m, 1H), 6.64 (d, J = 2.4 Hz, 1H), 6.21 (d, J = 2.4 Hz, 1H), 4.63 (s, 1H), 4.42–4.41 (d, J = 4.8 Hz, 2H), 3.66 (s, 3H), and 1.47 (s, 9H); ^13^C NMR (100 MHz,CDCl_3_) δ 162.93 (d, J = 243 Hz), 155.28, 138.62 (d, J = 7.9 Hz), 129.85 (d, J = 8.6 Hz), 125.19, 123.89, 123.53, 122.71, 114.68 (d, J = 21.4 Hz), 112.54 (d, J = 21 Hz), 107.50, 79.64, 34.55, 34.07, and 28.32; HRMS (ESI) calcd for C_17_H_21_FN_2_O_2_ [M + Na]^+^: 327.1479, found 324.1472.

tert-Butyl ((3-(3-bromophenyl)-1-methyl-1H-pyrrol-2-yl)methyl)carbamate (B5). White solid; mp 124–127°C; 40% yield; ^1^H NMR (400 MHz, CDCl_3_) δ 7.46 (s, 1H), 7.35–7.32 (m, 1H), 7.23–7.20 (m, 2H), 6.61 (d, J = 2.8 Hz, 1H), 6.17 (d, J = 2.8 Hz, 1H), 4.59 (s, 1H), 4.37 (d, J = 5.2 Hz, 2H), 3.62 (s, 3H), and 1.44 (s, 9H); ^13^C NMR (100 MHz, CDCl_3_) δ 155.25, 138.53, 130.89, 129.94, 128.70, 126.49, 125.23, 123.57, 122.75, 122.54, 107.50, 79.65, 34.52, 34.09, and 28.33; HRMS (ESI) calcd for C_17_H_21_BrN_2_O_2_ [M + H]^+^: 365.0859, found 365.0857.

tert-Butyl ((1-methyl-3-phenyl-1H-pyrrol-2-yl)methyl)carbamate (B6). White solid; mp 87–90°C; 46% yield; ^1^H NMR (400 MHz, CDCl_3_) δ 7.37–7.30 (m, 4H), 7.24–7.20 (m, 1H), 6.61 (d, J = 2.4 Hz, 1H), 6.20 (d, J = 2.4 Hz, 1H), 4.67 (s, 1H), 4.40 (d, J = 4.8 Hz, 2H), 3.62 (s, 3H), and 1.45 (s, 9H); ^13^C NMR (100 MHz,CDCl_3_) δ 155.26, 136.30, 128.41, 127.98, 125.73, 124.92, 122.43, 107.46, 79.40, 34.56, 33.91, and 28.24; HRMS (ESI) calcd for C_17_H_22_N_2_O_2_ [M + H]^+^: 287.1754, found 287.1750.

tert-Butyl ((1-methyl-3-(4-(trifluoromethyl)phenyl)-1H-pyrrol-2-yl)methyl)carbamate (B7). White solid; mp 120–122°C; 37% yield; ^1^H NMR (400 MHz, CDCl_3_) δ 7.62 (d, J = 8 Hz, 2H), 7.43 (d, J = 8.0 Hz, 2H), 6.67 (d, J = 2.8 Hz, 1H), 6.25 (d, J = 2.8 Hz, 1H), 4.66 (s, 1H), 4.42 (d, J = 4.8 Hz, 2H), 3.68 (s, 3H), and 1.47 (s, 9H); ^13^C NMR (100 MHz,CDCl_3_) δ 155.27, 140.06, 127.99, 127.74 (q, J = 32.3 Hz), 125.56, 125.43 (q, J = 3.8 Hz), 124.41 (q, J = 270 Hz), 123.70, 122.98, 107.63, 79.75, 34.64, 34.12, and 28.34; HRMS (ESI) calcd for C_18_H_21_F_3_N_2_O_2_ [M + H]^+^: 355.1628, found 355.1623.

tert-Butyl ((3-(2-fluorophenyl)-1-methyl-1H-pyrrol-2-yl)methyl)carbamate (B8). White solid; mp 81–84°C; 39% yield; ^1^H NMR (400 MHz, CDCl_3_) δ 7.31–7.27 (m, 1H), 7.25–7.21 (m, 1H), 7.17–7.08 (m, 2H), 6.68 (d, J = 2.4 Hz, 1H), 6.17 (d, J = 2.8 Hz, 1H), 4.81 (s, 1H), 4.29 (d, J = 5.6 Hz, 2H), 3.68 (s, 3H), and 1.45 (s, 9H); ^13^C NMR (100 MHz, CDCl_3_) δ 159.50 (d, J = 241.7 Hz), 155.45, 131.63 (d, J = 3.7 Hz), 127.78 (d, J = 8.3 Hz), 126.72, 124.19 (d, J = 3.5 Hz), 124.05 (d, J = 15.3 Hz), 122.71, 117.68, 115.60 (d, J = 23.3 Hz), 108.39, 79.36, 34.81, 34.21, and 28.35; HRMS (ESI) calcd for C_17_H_21_FN_2_O_2_ [M + H]^+^: 305.1660, found 305.1657.

### Tyrosinase Inhibitory Assay

The mushroom tyrosinase activity assays of synthetic compounds were carried out according to the previously reported modification (Qin et al., 2015). l-DOPA was used as the substrate. Briefly, 800 μl of phosphate buffer (50 mM, pH 6.8), 50 μl of mushroom tyrosinase (666.67 U/ml, dissolved in PBS), and 50 μl of inhibitor (dissolved in DMSO) were placed in the plastic centrifuge tube. Then, 100 μl of l-DOPA (5 mM, dissolved in PBS) was added. Subsequently, its change in absorbance at 475 nm was measured in a time-dependent manner using a Beckman UV-650 spectrophotometer. The percentage inhibition was calculated by using [(OD_0_ - OD_1_)/OD_0_] × 100%, where OD_0_ was the absorbance without inhibitor, and OD_1_ was the absorbance with the inhibitor. The IC_50_ value of the potential compound was calculated from dose-response curves of percentage inhibition. Kojic acid was used as a standard control for comparison. All experiments were performed in duplicate.

### Inhibition Mechanism and Kinetic Analysis

Experiments were performed to analyze the inhibitory mechanism by the following method. For the text, the mushroom tyrosinase at a final concentration of 16.67–100.00 U/ml and l-DOPA at a final concentration of 0.5 mM were chosen. Then, the mushroom tyrosinase activity was measured in the presence of the inhibitor.

The inhibition type of the inhibitor on mushroom tyrosinase was evaluated by the Lineweaver–Burk plot, and the inhibition constants were obtained from the second plots of the apparent 1/V_max_ and K_m_/V_max_ against the inhibitor concentration. In this assay, mushroom tyrosinase at a final concentration of 33.33 U/ml and l-DOPA at a final concentration of 0.25–2 mM were used. The equation for the Lineweaver–Burk plot can be written as follows:

$$\frac{1}{v}-\frac{K_{\text{m}}}{V_{\text{m}}}\left(1+\frac{\left[\text{I}\right]}{K_{\text{I}}}\right)+\frac{1}{V_{\text{m}}}\left(1+\frac{\left[\text{I}\right]}{K_{\text{IS}}}\right)$$

where v is the reaction velocity, K_m_ is the Michaelis constant, V_m_ is the maximal velocity, [I] is the concentration of the inhibitor, [S] is the concentration of the substrate, K_I_ is the constant for the inhibitor binding with the free enzyme, and K_IS_ is the constant for the inhibitor binding with the enzyme-substrate complex. K_I_ and K_IS_ were obtained from the slope or the vertical intercept vs. the inhibitor concentration, respectively.

### Molecular Docking Study

Sybyl 2.1.1 (Tripos, United States) was used for the docking simulations between mushroom tyrosinase and the inhibitor. First, compound A12 was prepared by the energy minimization using the MM2 program, with energy termination of 0.01 kcal/mol, iteration of 1,000 times, and charges of Gasteiger-Hückle. Second, the crystal structure of mushroom tyrosinase (PDB ID: 2Y9X) was prepared by extracting ligands, removing H_2_O, termini treatment, and adding hydrogens. The active pocket of tyrosinase was generated using the ligand mode. Finally, the docking simulation between tyrosinase and compound A12 was carried out in the default format. PyMOL software was used to draw the view based on the Sybyl results.

### Cell Culture

B16 mouse melanoma cells were cultured in Dulbecco’s modified Eagle’s medium (DMEM) containing 10% fetal bovine serum and 1% penicillin/streptomycin at 37°C in a 5% CO_2_ incubator.

### MTT Assay

B16 cells (100 µl) were plated in a 96-well plate (5 × 10^3^ cells/well) for 24 h and then treated with a 100 µl complete medium containing A12 or kojic acid for 24 h. MTT solution (0.5 mg/ml, final concentration) was added to each well, and plates were incubated at 37°C for another 3 h. The absorbance was measured at 490 nm using a multi-detection microplate reader.

### Cellular Tyrosinase Activity

The inhibitory activity of A12 on tyrosinase in B16 cells was evaluated. B16 cells (5 × 10^4^ cells/well) were plated in 96-well plates and incubated for 24 h. A measure of 100 µl of complete medium including A12 or kojic acid was added for further incubation of 48 h. Then, cells were added to 50 µl Triton X-100 (1%) and allowed for freezing for 1 h at −80°C. After thawing at room temperature, 10 μl l-DOPA solutions (0.1%, w/v) were added and co-incubated for 1 h at 37°C. The absorbance at 405 nm was measured.

## Results and Discussion

### Chemistry

**Scheme 1:**
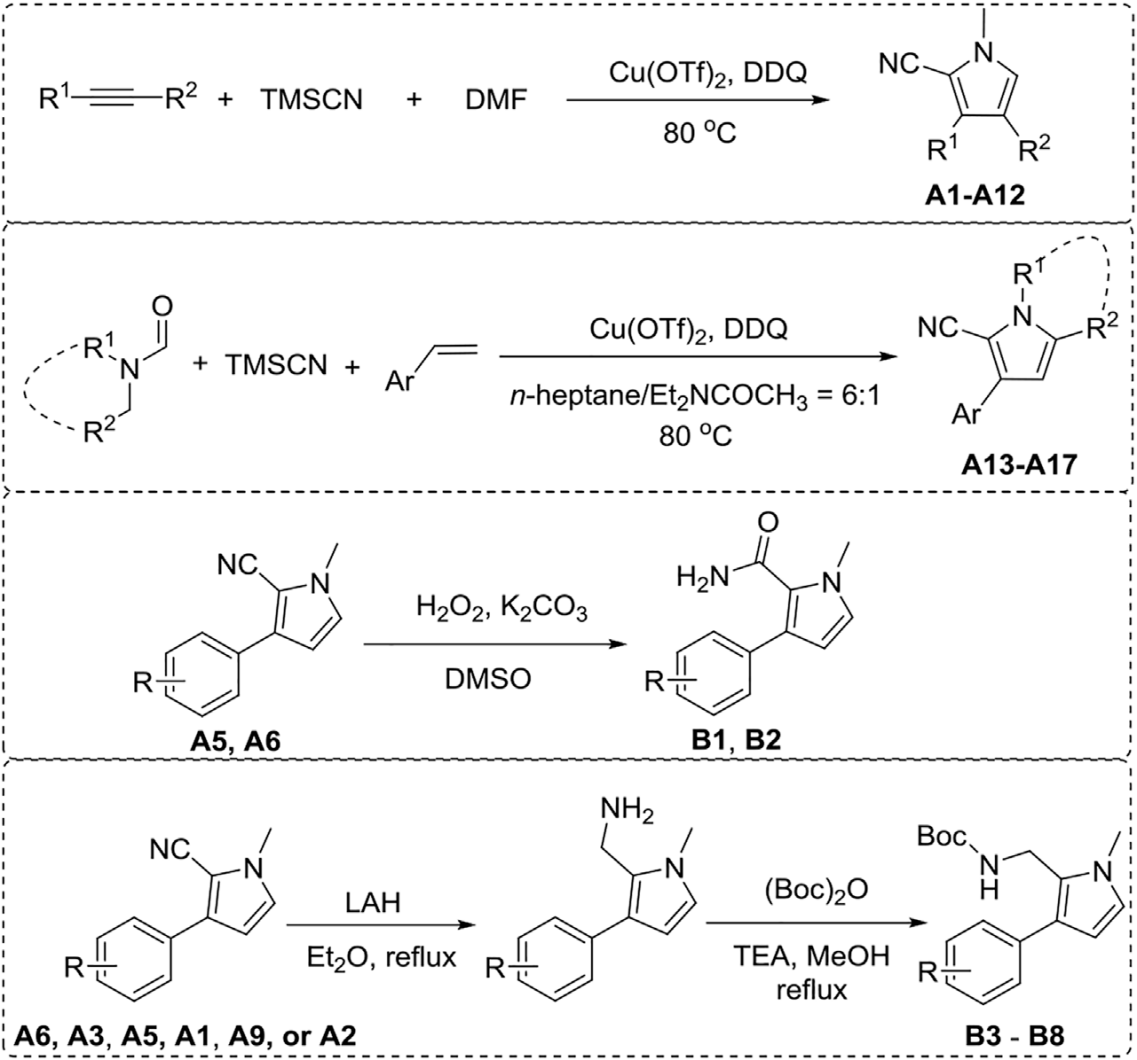
General synthesis of A (1–17) and B (1–8).

Pyrrole derivatives A (1–17) and B (1–8) were synthesized according to the synthetic route shown in Scheme 1. A1–A12 were obtained using aromatic olefin or alkyne, DMF, and TMSCN as starting materials under the catalysis of Cu(OTf)_2_ and DDQ. A (13–17) were synthesized from aromatic olefin, N,N-disubstituted formamide, and TMSCN with Cu(OTf)_2_ as the catalyst and DDQ as additive. B 1–2 could be synthesized from compound A5 or A6 through a hydrolysis reaction. Meanwhile, the reduction of compounds A6, A3, A5, A1, A9, or A2 by LAH with subsequent amine derivatization by (Boc)_2_O would produce B (3–8). ^1^H NMR, ^13^C NMR, and HRMS were applied to confirm their structures.

### Tyrosinase Inhibitory Activity Assay and SAR Analysis

Tyrosinase inhibitory assay of all synthesized pyrrole derivatives A (1–17) and B (1–8) was performed using l-DOPA as the substrate. As shown in Table 1, compounds A (1–17) exhibited moderate to excellent inhibitory activities against tyrosinase, with IC_50_ values ranging from 0.97 to 89.15 μM. In particular, A12 (IC_50_ = 0.97 μM) showed the strongest inhibitory activities, which were ∼30 times stronger than the reference inhibitor kojic acid (IC_50_ = 28.72 μM).

**TABLE 1:** Inhibitory activity of N-heterocycle derivatives on tyrosinase.

Compound	Structure	IC_50_ (μM)	Compound	Structure	IC_50_ (μM)
A1	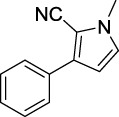	7.42	A2	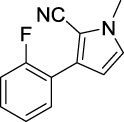	8.72
A3	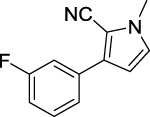	21.43	A4	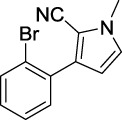	8.47
A5	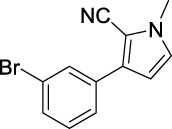	16.52	A6	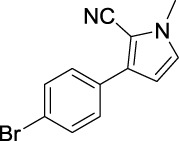	8.17
A7	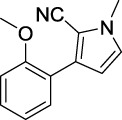	23.57	A8	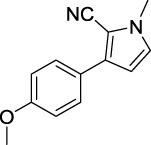	89.15
A9	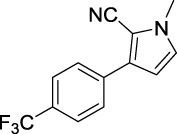	12.44	A10	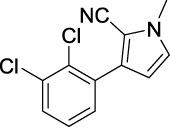	4.83
A11	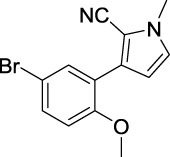	2.11	A12	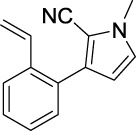	0.97
A13	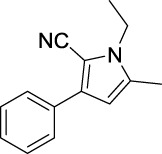	4.46	A14	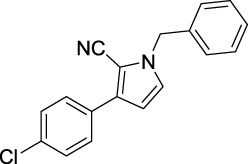	80.46
A15	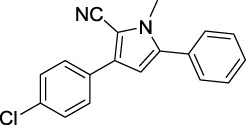	87.32	A16	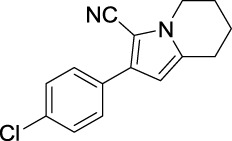	5.06
A17	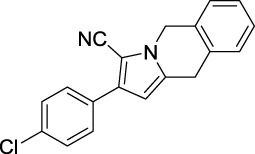	5.57			
B1	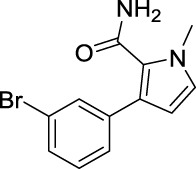	>200	B2	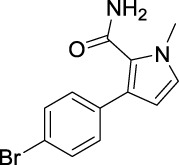	>200
B3	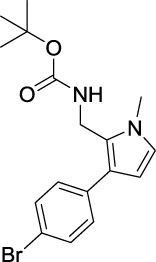	>200	B4	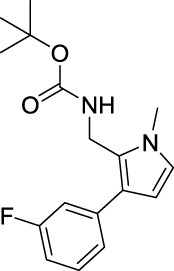	>200
B5	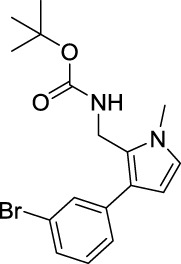	>200	B6	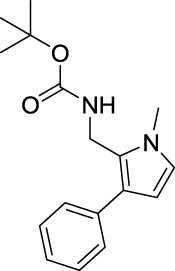	>200
B7	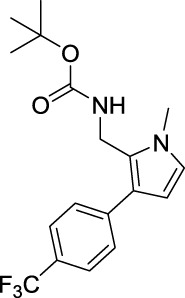	>200	B8	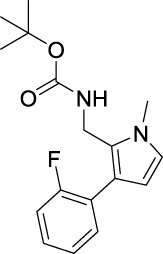	>200
Kojic acid		28.72			

Based on the data displayed in Table 1, the SAR of the tested compounds against tyrosinase was analyzed. For compounds A (1–17), compound A1 (IC_50_ = 7.24 μM) with no substitute group was selected as the template compound. It was clear that the introduction of a substituent on the phenyl ring at the 3-position of the pyrrole skeleton did show the clear influence of the corresponding inhibition potency. Among them, compound A2 with the 2-fluorine group on the benzene ring (IC_50_ = 8.72 μM), A3 with the 3-fluorine group (IC_50_ = 21.43 μM), A4 with the 2-bromine group (IC_50_ = 8.47 μM), A5 with the 3-bromine group (IC_50_ = 16.52 μM), A6 with the 4-bromine group (IC_50_ = 8.17 μM), A7 with the 2-methoxy group (IC_50_ = 23.57 μM), A8 with the 4-methoxy group (IC_50_ = 89.15 μM), and A9 with the 4-trifluoromethyl group (IC_50_ = 12.44 μM) all presented lower inhibitory activities than compound A1. These results indicated that the introduction of fluorine, bromine, methoxy and trifluoromethyl alone might cause a decrease in tyrosinase inhibitory activity. In contrast, two compounds A10 and A11 with dual substituents on the benzene ring provided better inhibitory results than compound A1 and showed an IC_50_ value of 4.83 and 2.11 μM, respectively. Moreover, A12 with a 2-vinyl group on the benzene ring (IC_50_ = 0.97 μM) also appeared to have much higher inhibitory activities than compound A1. This result revealed the special property of the 2-vinyl group during the interaction between A12 and tyrosinase. As for the substituent on the 1- and 5-positions of the pyrrole ring, additional five compounds A (13–17) were further tested. Based on the results, it is obvious that the presence of an untethered benzyl at 1-position (A14) or the phenyl group at 5-position (A15) would dramatically affect the inhibitory activities. On the contrary, A13 with the ethyl group on nitrogen of the pyrrole ring (IC_50_ = 4.46 μM), A16, and A17 with the pyrrole as a part of a fused ring system showed higher inhibitory activities (IC_50_ = 5.06 and 5.57 μM, respectively) than compound A1, which revealed that the alkyl group at 1-position or a planar type of the molecule might increase the inhibitory activity against tyrosinase. Therefore, based on the previous SAR results of all compounds, further derivatization of A1 with other ring systems at 3-position of the pyrrole ring or other fused ring systems will be designed and screened.

For investigating the effect of the cyano group on the inhibitory activities, it was transformed into an amide group (B1, B2) or carbamate group (B3 ∼ 8), and the inhibitory activities of corresponding derivatives were obviously reduced. These results indicated that the cyano group of compounds A1-A17 was crucial for tyrosinase inhibition.

### Inhibition Mechanism

**FIGURE 1:**
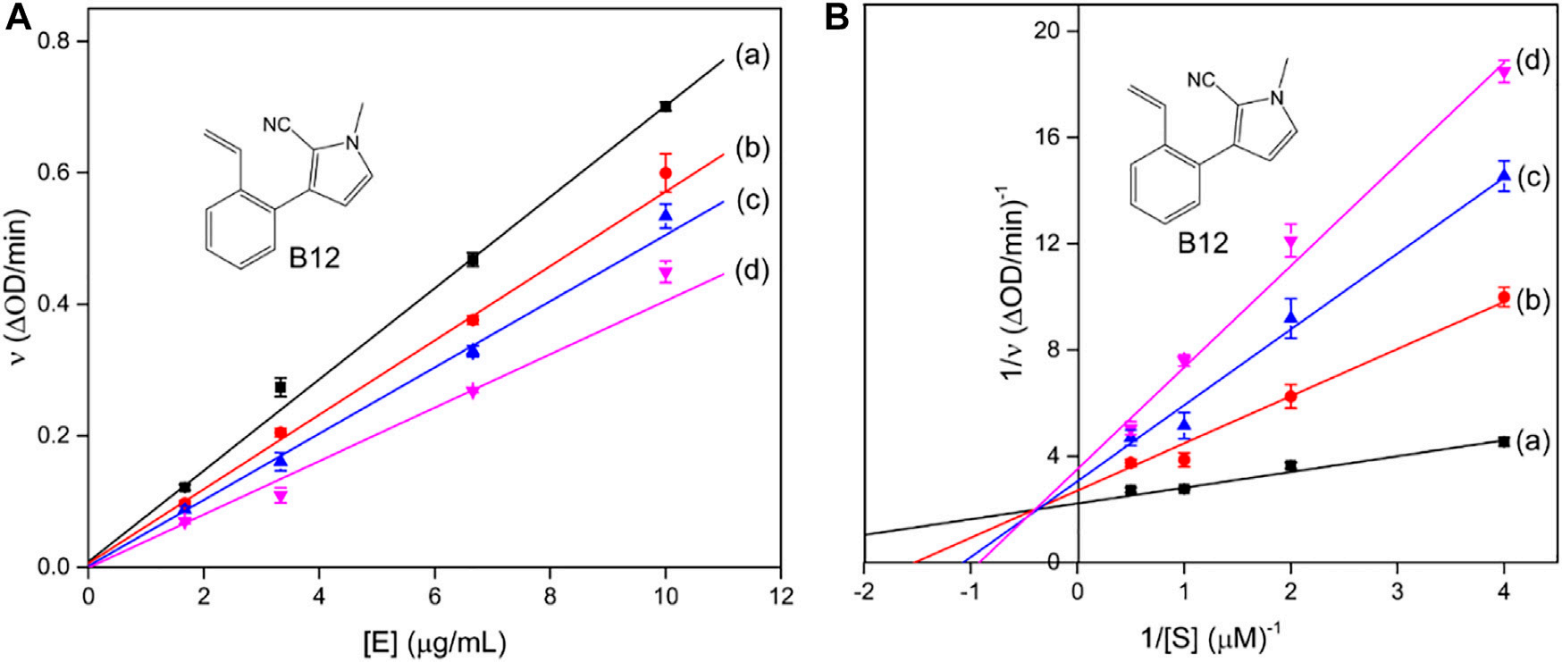
Plots of ν vs. [E] and the Lineweavere–Burk plot of compound A12 (concentrations: (a) 0, (b) 0.75, (c) 1 μM, and (d) 1.5 μM).

Compound A12 with the best inhibition activity was selected as the lead compound to ascertain the inhibition mechanism of mushroom tyrosinase. Figure 1A shows the plots of initial velocity vs. tyrosinase concentrations at different concentrations of compound A12. As can be seen, the plots gave a family of straight lines, respectively, which passed through the origin point. Moreover, the slopes of the lines extended a descent with the increase of concentrations of compound A12. These results revealed that the presence of compound A12 did not bring down the amount of active enzyme but just resulted in the reduction of the enzyme activity. Hence, the tyrosinase inhibition of compound A12 was reversible.

**FIGURE 2:**
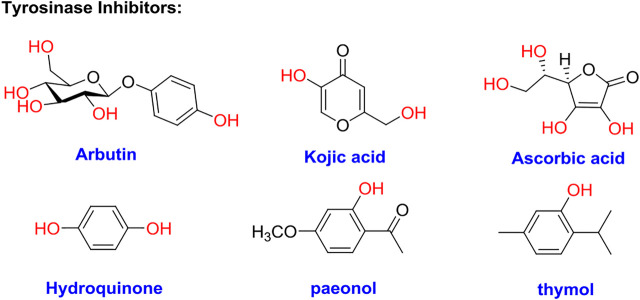
Representative tyrosinase inhibitors.

To further explore the inhibition kinetic behavior of the most promising compound A12 on mushroom tyrosinase, the tyrosinase activity was determined under different concentrations of l-DOPA in the presence or absence of an inhibitor. The results were analyzed using Lineweaver–Burk double reciprocal plots. For compound A12 (Figure 2B), the results presented that the plots of 1/ν vs. 1/[S] gave straight lines with different slopes intersecting one another in the second quadrant. The values of V_m_ and K_m_ all descended with the increase in concentration of A12, which suggested that compound A12 induced mixed-type inhibition of mushroom tyrosinase. In other words, compound A12 could bind with both free enzyme and the enzyme-substrate complex, which was similar to the inhibition type of kojic acid. Hence, we determined the equilibrium constant for inhibitor binding with free enzyme (K_I_) and the enzyme-substrate complex (K_IS_) through the second plots of the apparent K_m_/V_max_ and 1/V_max_ vs. inhibitor concentrations. The K_I_ and K_IS_ values of the compound A12 were calculated as 1.11 and 1.00 μM.

### Molecular Docking

**FIGURE 3:**
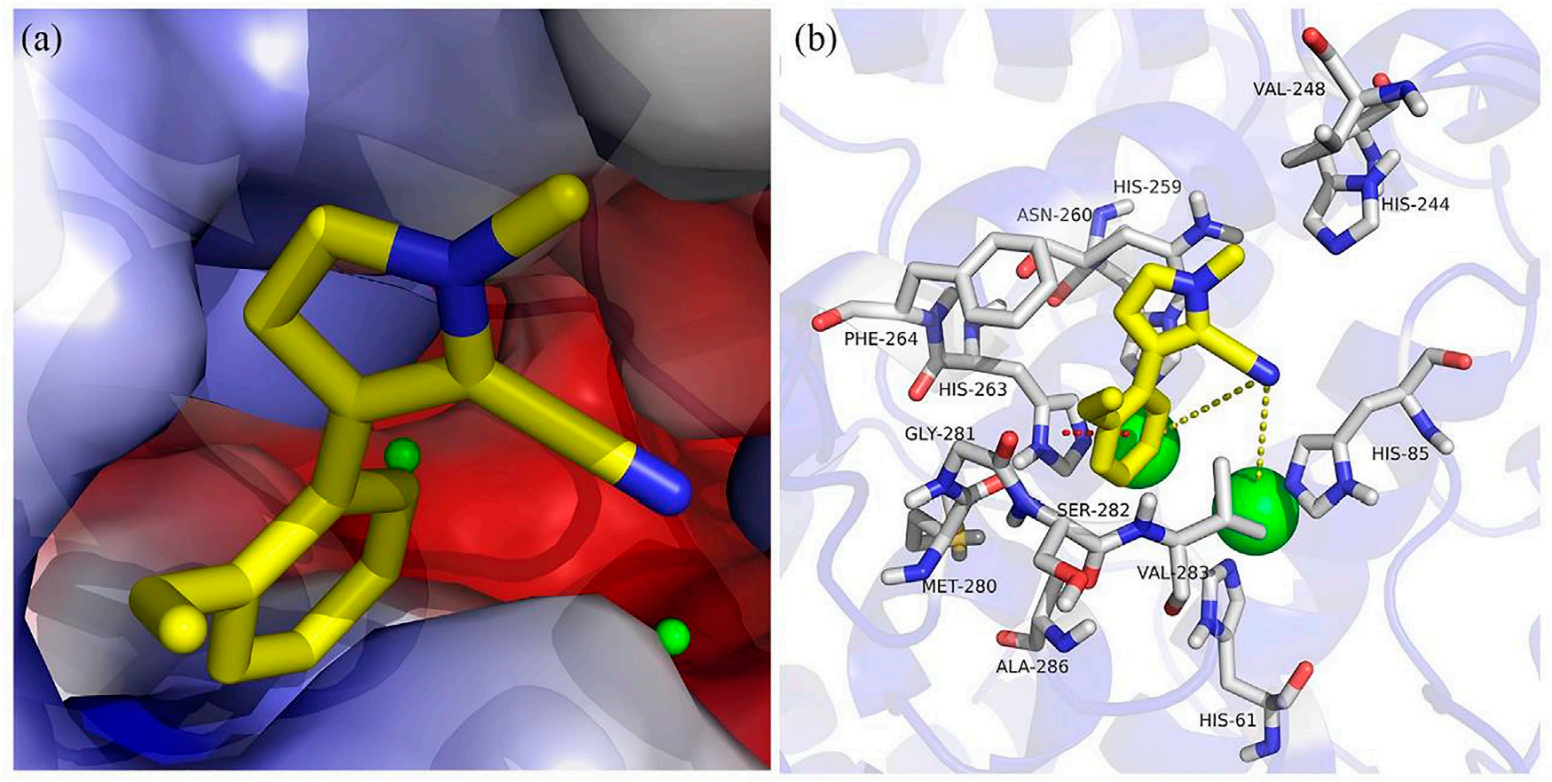
Molecular docking of compound A12 with tyrosinase: **(A)** in the active pocket; **(B)** interactions with amino acid residues.

To better understand the inhibitory activity of compound A12, Sybyl and PyMOL software were conducted to understand the interaction pattern between the A12 and the active site of tyrosinase. Mushroom tyrosinase (PDB: 2Y9X) from the RCSB Protein Data Bank was selected as the target protein for simulation. The docking simulation results showed that A12 was well accommodated and bound in the active pocket of tyrosinase (Figure 3A) and had in-depth interactions with the active site residues (Figure 3B). The cyano group of A12 resided adjacent to the dicopper nucleus, indicating that there was a potential metal-ligand interaction, which was considered to be one of the key interactions between tyrosinase and the ligand compound. The pyrrole ring formed a π-π interaction with His263. Furthermore, A12 formed hydrophobic interactions with Ala286, Asn260, Gly281, His244, His259, Met280, Phe264, Ser282, Val248, and Val283.

### Effect of Compound A12 on Cellular Tyrosinase Activity

**FIGURE 4:**
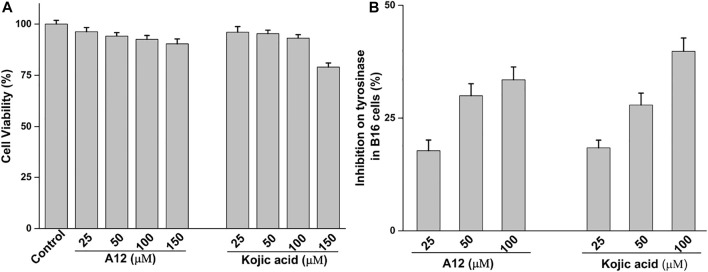
**(A)** Cell cytotoxicityof A12 and kojic acid **(B)** and inhibition of cell tyrosinase activity of A12.

For evaluating the effect of compound A12 on cellular tyrosinase activity, its cell cytotoxicity was first tested. The results showed that compound A12 and kojic acid showed no cytotoxicity on B16 melanoma cells under a concentration of 100 μM (Figure 4A). Thence, the concentration of A12 (under 100 μM) was selected for the assay of the cellular tyrosinase activity. A12 could effectively inhibit the tyrosinase activity in B16 melanoma cells in a dose-dependent manner (Figure 4B) and present an effective inhibitory of 33.48% at a concentration of 100 μM, which was equivalent to that of Kojic acid (39.81%).

## Conclusion

To conclude, the inhibitory activities of two series of pyrrole derivatives A (1–17) and B (1–8) were screened against tyrosinase. Compound A12 showed the highest inhibition activity with reversible and mixed-type inhibition types. Compound A12 (IC_50_: 0.97 μM) showed ∼30 times stronger inhibition activity than the reference inhibitor kojic acid (IC_50_: 28.72 μM). Molecular docking studies showed that the metal–ligand interaction, π-π interaction, and hydrophobic interactions played important roles in the interaction between tyrosinase and A12. Furthermore, A12 (100 μM) presented effective inhibitory on tyrosinase in B16 melanoma cells with an inhibition rate of 33.48%, which was equivalent to that of kojic acid (39.81%). Our studies have indicated that these pyrrole derivatives have the potential to be developed as anti-tyrosinase agents for use in medicine, agriculture and food industry, and cosmetic products.

## Data Availability

The original contributions presented in the study are included in the article/Supplementary Material; further inquiries can be directed to the corresponding authors.
